# The Effect of Cognitive Task, Gait Speed, and Age on Cognitive–Motor Interference during Walking

**DOI:** 10.3390/s23177368

**Published:** 2023-08-24

**Authors:** Jessica Pitts, Kunal Singhal, Yashashree Apte, Prakruti Patel, Lakshmi Kannan, Tanvi Bhatt

**Affiliations:** 1 Department of Physical Therapy, University of Illinois at Chicago, 1919 W Taylor St., Chicago, IL 60612, USA; 2 Department of Physical Therapy, University of St. Augustine for Health Sciences, Austin, TX 32086, USA; 3 Department of Health and Exercise Science, Colorado State University, Fort Collins, CO 80521, USA

**Keywords:** dual-tasking, aging, cognitive–motor interference, gait, prioritization

## Abstract

Dual-tasking can cause cognitive–motor interference (CMI) and affect task performance. This study investigated the effects of age, gait speed, and type of cognitive task on CMI during gait. Ten younger and 10 older adults walked on a pressure-sensitive GAITRite walkway which recorded gait speed and step length. Participants walked at a slow, preferred, or fast speed while simultaneously completing four cognitive tasks: visuomotor reaction time (VMRT), serial subtraction (SS), word list generation (WLG), and visual Stroop (VS). Each combination of task and speed was repeated for two trials. Tasks were also performed while standing. Motor and cognitive costs were calculated with the formula: ((single-dual)/single × 100). Higher costs indicate a larger reduction in performance from single to dual-task. Motor costs were higher for WLG and SS than VMRT and VS and higher in older adults (*p* < 0.05). Cognitive costs were higher for SS than WLG (*p* = 0.001). At faster speeds, dual-task costs increased for WLG and SS, although decreased for VMRT. CMI was highest for working memory, language, and problem-solving tasks, which was reduced by slow walking. Aging increased CMI, although both ages were affected similarly by task and speed. Dual-task assessments could include challenging CMI conditions to improve the prediction of motor and cognitive status.

## 1. Introduction

Activities of daily living often involve walking while simultaneously performing an attention-demanding cognitive task, such as talking on the phone (i.e., dual-tasking). Dual-tasking can result in a deterioration in performance on either the cognitive or motor task (or both) due to limited cognitive resources, known as cognitive–motor interference (CMI) [1,2]. Four main CMI patterns emerge when performing a motor and cognitive task: (1) motor-related cognitive interference (motor prioritized and cognitive performance deteriorates), (2) cognitive-related motor interference (cognitive prioritized and motor performance deteriorates), (3) mutual interference (both tasks prioritized, performance on both deteriorate), and (4) mutual facilitation (both tasks prioritized and performance on both improves) [1,3]. Multiple CMI patterns occur during dual-task gait [1,4,5], which may result from differences in dual-task protocols or conditions between studies. There is evidence that CMI can be affected by both the type of cognitive task (e.g., mental math, reaction time, and verbal fluency) and gait speed [6,7]. However, there is a limited understanding of how the combination of these factors affect CMI, which limits the translatability of dual-task gait protocols into clinical practice.

CMI during gait depends on the type of cognitive task [6]. Specifically, studies have shown that performing a serial subtraction task during walking affected motor performance (quantified by gait speed, trunk control, and stride time variation) more than a Stroop task, verbal fluency task, or listening to a story [8,9,10]. Additionally, the type of cognitive task may affect task prioritization. Patel, Lamar, and Bhatt (2014) showed that young adults prioritized their cognitive performance when completing a Stroop task but contrastingly prioritized their gait speed during a reaction time task [7]. These results suggest that tasks demanding high levels of attention (e.g., mental math and Stroop) may result in cognitive prioritization and motor deterioration. However, very few studies have compared CMI between more than two different cognitive tasks, and the relationship between cognitive demand and CMI during dual-task gait remains unclear.

Alterations in gait speed can also change the attentional demands of walking and affect CMI. One study showed that while walking slowly, young adults performed better on a Stroop task (i.e., more correct responses) compared to preferred gait speed [7]. However, walking too slowly may have the opposite effects; one study showed that walking very slowly (20% of preferred speed) while dual-tasking resulted in greater cognitive costs [11]. Further, Dennis et al. [12] showed that fast walking led to more errors on a cognitive task than preferred walking in individuals with stroke. Therefore, a slight slowing of gait may lower the attentional demands of walking due to increased stability or reduced postural threat [13], while larger alterations from normal walking speed (i.e., fast or extremely slow walking) may increase CMI. However, the existing understanding of how walking speed affects CMI is limited because related studies have primarily focused on young adults and the modulatory effect of walking speed on CMI in older adults has been left largely unexamined.

Investigating how cognitive task type and gait speed affect CMI in older adults could inform the development of dual-task gait protocols for clinical implementation, which is a promising tool for accurate, low-cost, and rapid prediction of cognitive and motor status in older adults. Dual-task costs (i.e., deterioration in performance from single-task to dual-task) are a strong predictor of falling in older adults [14] and can better indicate fall risk than single-task gait parameters alone [15,16]. Further, dual-task assessments can indicate cognitive impairment with higher sensitivity than conventional cognitive assessments [17]. However, dual-task gait assessments have primarily been employed in research settings, and their clinical translation is still in a relatively novel stage [16]. This could be due to a lack of protocol standardization, as there is a lack of information on which specific cognitive tasks (e.g., type and difficulty) and walking conditions (e.g., gait speed) could provide the most accurate assessment while maintaining the highest level of safety. For example, clinicians may want to initially implement lower CMI conditions to maintain safety and progress to more cognitively demanding conditions after improvement or repetition.

The purpose of this study was to investigate the effects of age (younger vs. older adults), gait speed (slow, preferred, fast), and type of cognitive task (visuomotor reaction time (VMRT), word list generation (WLG), serial subtraction (SS), and visual Stroop (VS)) on motor and cognitive costs during dual-task gait. We hypothesized that SS and VS would lead to higher CMI than other tasks. We also hypothesized that slow walking would enhance cognitive performance, whereas fast walking would diminish cognitive performance. Lastly, we hypothesized that older adults would have higher CMI than young adults and may be differently affected by task and speed.

This study will inform which cognitive tasks and walking speeds induce the most CMI and thus might be the most effective predictors of cognitive or motor status for older adults in clinical dual-task gait assessments. Further, this study will provide a deeper insight into the attentional demands of walking in real-life multi-tasking situations, which involve numerous types of cognitive tasks (e.g., mentally calculating the cost of groceries, mapping a route to a destination and carrying out a conversation) and changes in gait speed (e.g., running behind schedule, walking slowly and talking to someone).

## 2. Materials and Methods

### 2.1. Participants

Twenty healthy participants, 10 younger and 10 older adults, were included in this study. Participants were classified as young adults if they were between the ages of 18–35 years old and were classified as older adults if they were between the ages of 60–90 years old. Specifically, younger adults who were included in the study ranged from ages 21–33, and older adults ranged from ages 61–76. Participants were excluded from the study if they had any self-reported neurological, musculoskeletal, cardiopulmonary, or other systemic disorders that may have affected their ability to participate in the study. Participants were also excluded if their weight was greater than 220 pounds to minimize the effect of weight/obesity on gait parameters. All older adults had intact cognitive functioning, indicated by a score ≥27 out of 30 on the Montreal Cognitive Assessment (MoCA), which is higher than the cutoff score for mild cognitive impairment (i.e., 26) [18]. To assess normal walking function in older adults, this group also completed the six-minute walk test, which involves continually walking for 6 min without any assistive device or shortness of breath. Older adults walked approximately 434.3 ± 137.8 m over six minutes, which is similar to the normative score for the six-minute walk test in older adults (344 m) [19] and indicates intact walking ability and physical function. Demographic details can be found in Table 1. This study was approved by the institutional review board at the University of Illinois at Chicago and informed consent was obtained for all participants.

### 2.2. Experimental Protocol

Participants completed single and dual-task trials while walking at a self-selected slow, preferred, or fast speed over a walkway. These speeds were self-selected and not controlled for because we wanted to mimic real-life conditions as much as possible and avoid interfering with participants’ natural walking speed and gait parameters. Additionally, this would allow us to compare differences in self-selected gait speeds between single and dual-task trials to calculate dual-task motor costs. Participants first completed one familiarization walking trial at their preferred speed to become accustomed to the walkway before beginning recorded trials. Participants completed single-task trials in all three walking speeds (slow, preferred, and fast), during which they walked while completing no additional cognitive task. In dual-task trials, participants completed four different cognitive tasks while walking. Participants were not asked to prioritize either task or were given generic, standardized instructions for every trial. For example, in a dual-task trial performing the serial subtraction task and walking at a slow speed, participants were given the instruction to “Walk at a slower pace than your preferred walking speed while you complete the serial subtraction task.” Each cognitive task was completed at each walking speed and all trials were completed twice. We asked participants to perform only two trials in each condition because we wanted to prevent the onset of mental/physical fatigue, which can have rapid onset [20,21] and negatively impact both cognitive and motor performance [22,23,24], therefore, confounding the dual-task effect. The order of these trials was randomized; thus, some participants completed the single-task condition prior to the dual-task condition and vice versa. Prior to these walking trials, all cognitive tasks were also completed in a randomized order while standing to determine cognitive single-task performance. Participants received standard instructions about how to perform the cognitive tasks and completed a familiarization trial before their responses were recorded. Mean values for performance on each cognitive task can be found in Table 2.

#### 2.2.1. Cognitive Tasks

Visuomotor Reaction Time Task (VMRT): Participants were shown a screen on which two stimuli would flash. A red signal would initially flash, followed by a green signal at an unexpected time. Participants were provided with a push button in their hand and asked to press the button as soon as they saw the green signal appear on the screen. To maintain hand position, participants were asked to hold the push button with their arm by their side and their elbow flexed at 90°. For this task, cognitive performance was determined by the amount of time (in milliseconds) between the presentation of the green signal and when the button was pushed. The screen with the visual stimuli was placed 1.5 m past the walkway, so as not to interfere with the normal walking pattern recorded on the walkway. Before beginning each trial, the researcher ensured that the participant could clearly see the screen from the starting position.

Word List Generation (WLG): Participants were given a letter (e.g., “F”) and asked to list words that started with this letter for 10 s. Cognitive performance was determined by the number of correct responses [25].

Serial Subtraction (SS): Participants were given a two-digit number and instructed to count backward by a given one-digit number for 10 s. Cognitive performance was determined as the number of correct responses [26].

Visual Stroop (VS): Names of colors were shown printed in different color inks on a screen. Participants were asked to name the color in which the word was printed, rather than read the word. For example, the correct response for the word “red” printed in green ink would be “green.” Twenty-five words were listed on the screen (placed 1.5 m past the walkway) at once and participants were asked to read the color of the ink of each word in sequential order. It was ensured that participants could read the font size of the printed words from their starting position before beginning the trial. This task was completed for 10 s and cognitive performance was determined by the number of correct responses [27].

**Figure 1:**
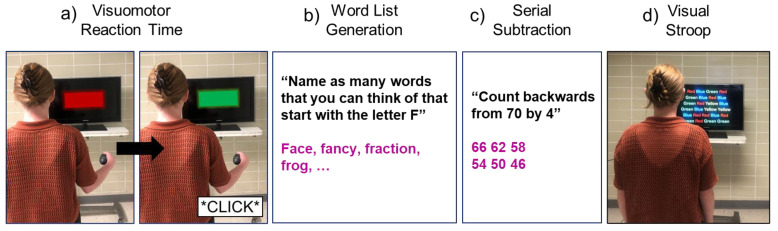
Cognitive tasks are performed in single-task standing. In the visuomotor reaction time task (**a**), participants stood in front of a TV screen and held a push button in their hand with their elbow flexed to 90°. A red signal or a green signal would flash on the screen, and participants were told to press the button as fast as possible when they saw the green signal appear, as indicated by the *click* in the second image. In the word list generation task (**b**), participants were given a letter (e.g., F) and asked to list as many words as possible which started with that letter (e.g., face, fancy, fraction, frog, etc.). In the serial subtraction task (**c**), participants were given a two-digit number (e.g., 70) and asked to count backward by a given one-digit number (e.g., four). For the word list generation and serial subtraction, an example of a researcher-given cue is indicated in black and an example of the participant response is indicated in purple. Lastly, in the visual Stroop task (**d**), participants were shown a screen with a series of names of colors printed in different colored inks. The participant was asked to read aloud the color of the text in which each word was printed in.

A visual representation of all cognitive tasks can be found in Figure 1.

#### 2.2.2. Motor Performance

**Figure 2:**
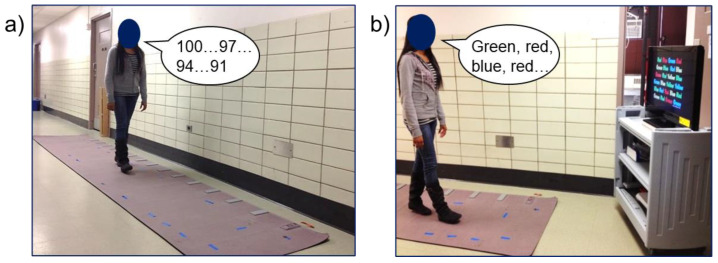
Dual-task gait set-up using the GAITRite pressure-sensitive mat. Participants would walk along the 12 ft mat at either a slow, preferred, or fast gait speed. Cognitive tasks were also performed simultaneously, including the visuomotor reaction time, word list generation, serial subtraction, and visual Stroop. (**a**) shows an image of a participant completing the serial subtraction task while walking and (**b**) shows an image of a participant completing the Stroop task while walking. Examples of correct participant responses for each task are indicated in black speech bubbles.

We evaluated motor performance using both gait speed and step length. Many previous studies have reported changes in both of these variables during dual-tasking [28,29,30,31,32], although most dual-task gait studies have focused on changes in gait speed [33]. Therefore, we wanted to examine the modulatory effect of age, task, and speed not only on motor costs for gait speed but also on underlying gait parameters (i.e., step length). Further, different cognitive tasks may not necessarily have the same modulatory effect on both gait speed and step length. A meta-analysis reported that the greatest dual-task costs for gait speed were observed for verbal fluency tasks, although the greatest dual-task costs for step length were observed for working memory tasks [7,28,33,34]. Gait parameters were recorded using an electronic GAITRite mat (CIR Systems, Inc., Sparta, NJ, USA), which is a 12 × 2 ft, pressure-sensitive mat capable of measuring spatio-temporal gait parameters using the accompanying GAITRite software. For a visual representation of the GAITRite mat and dual-task gait experimental set-up, refer to Figure 2.

To ensure that participants maintained a steady state gait pattern, they were instructed to start walking one meter before the mat and stop walking once they reached one meter past the other end of the mat. Gait speed was calculated as the length of the mat (12 ft) divided by the time taken to walk across the mat for that specific trial. Step length was calculated as the distance between the heel center of one footprint to the heel center of the previous footprint on the opposite limb and averaged for all steps along the mat for that specific trial. We then normalized step length to each participant’s body height. Mean gait speed and step length in each single and dual-task condition can be found in Table 3.

### 2.3. Sample Size Calculation

We conducted an a priori power analysis based on data available from our previous related study [7] in young adults to calculate the sample size necessary to detect significant differences in dual-task costs between cognitive tasks and gait speeds. With a significance level of 0.05 and power of 0.80, the sample size necessary to detect differences between these factors was calculated as *n* = 8 in each group. Thus, a conservative sample size of 10 in each group was selected for the present study. The power analysis was conducted using G*Power version 3.1.

### 2.4. Statistical Analysis

To ensure that participants walked at different speeds based on the given instruction (slow, preferred, or fast), we conducted a one-way ANOVA to test the effect of gait speed instruction on actual gait speed for younger and older adults. To compare the effects of dual-tasking on gait and cognition between age groups, cognitive tasks, and gait speeds, we calculated motor and cognitive costs. This was conducted by comparing motor (gait speed and step length) and cognitive performance (reaction time (VMRT) or number of correct responses (SS, WLG, and VS)) between single and dual-task using the following formula: (single-dual)/single × 100 [35]. Higher motor and cognitive costs indicate that there was a larger reduction in performance from single-task to dual-task. Specifically, higher gait speed and step length motor costs indicate that there was a larger reduction in gait speed or step length (respectively) during dual-task compared to single-task walking. Similarly for cognitive costs, higher costs indicate that there was a larger decline in performance during dual-task compared to single-task, either indicating increased reaction time (VMRT) or reduced number of correct responses (WLG, SS, and VS). Motor or cognitive costs close to zero indicate that performance was not very different between single-task and dual-task.

A 2 × 3 × 4 mixed design ANOVA was used to analyze the main effects and interaction effects of age (young and old), speed (slow, preferred, and fast), and cognitive task (VMRT, WLG, SS, and VS) on both motor and cognitive costs. The Bonferroni correction was used to conduct post hoc tests. We used Mauchly’s test of sphericity to test the assumption of sphericity prior to ANOVA testing. If the sphericity assumption was not met, the Greenhouse–Geisser correction was used. The level of significance was set at 0.05 for all tests.

## 3. Results

An initial ANOVA indicated that gait speed was significantly different between all instructions (slow, preferred, and fast) for both young adults (F(2,18) = 49.523, *p <* 0.001, η^2^p = 0.846) and older adults (F(2,18) = 88.559, *p* < 0.001, η^2^p = 0.908).

### 3.1. Main Effect of Task

**Figure 3:**
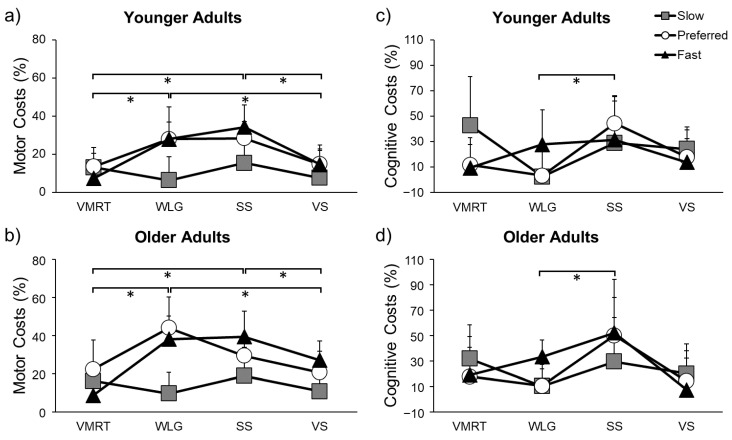
Motor costs for gait speed (**a**,**b**) and cognitive costs (**c**,**d**) during dual-task gait. Younger and older adults performed the visuomotor reaction time (VMRT), word list generation (WLG), serial subtraction (SS), and visual Stroop (VS) tasks while walking at their slow, preferred, and fast speeds. Higher costs indicate a larger reduction in performance from single-task to dual-task. * indicates a significant difference in costs (*p* < 0.05) between tasks.

The task effect on gait speed motor costs (F(3,54) = 24.155, *p* < 0.001, η^2^p = 0.573), step length motor costs (F(3,54) = 12.550, *p* < 0.001, η^2^p = 0.411), and cognitive costs (F(3,54) = 6.005, *p* = 0.001, η^2^p = 0.273) was significant. As can be seen in Figure 3, gait speed motor costs were significantly higher for WLG and SS compared to VMRT (*p* < 0.001 for both) and VS (*p* < 0.001 for both). The same was seen for step length motor costs, which were significantly higher for WLG and SS compared to VMRT (*p* = 0.038; *p* = 0.034) and VS (*p* < 0.001 for both). This indicates that there was a greater slowing of gait speed and reduction in step length from single-task parameters while performing the WLG and SS compared to the VMRT and VS. Due to the similarity of dual-task motor costs for gait speed and step length, only motor costs for gait speed are graphically presented in the manuscript. 

Cognitive costs were significantly higher for SS than WLG (*p* = 0.009), which indicates that there was a larger reduction in the number of correct responses from single-task to dual-task on the SS compared to the WLG.

### 3.2. Main Effect of Speed

The speed effect on gait speed motor costs (F(2,36) = 18.852, *p* < 0.001, η^2^p = 0.512) and step length motor costs (F(1.453,26.151) = 14.964, *p* < 0.001, η^2^p = 0.454) was significant. Both gait speed and step length motor costs were lower at slow speed compared to preferred (speed: *p* < 0.001; step length: *p* = 0.002) and fast speeds (speed: *p* = 0.001; step length: *p* = 0.001). Thus, gait speed and step length were more similar between single and dual-task at slow speeds. The speed effect on cognitive costs was non-significant (F(2,36) = 0.525, *p* = 0.597).

### 3.3. Main Effect of Group

The group effect on gait speed motor costs was significant (F(1,18) = 4.880, *p* = 0.040, η^2^p = 0.213). Motor costs were significantly higher in older adults compared to young adults, which indicates that older adults had a larger reduction in performance when dual tasking compared to younger adults. The group effect on step length motor costs (F(1,18) = 1.768, *p* = 0.200) and cognitive costs (F(1,18) = 2.073, *p* = 0.169) was non-significant.

### 3.4. Task X Speed Interaction Effect

**Figure 4:**
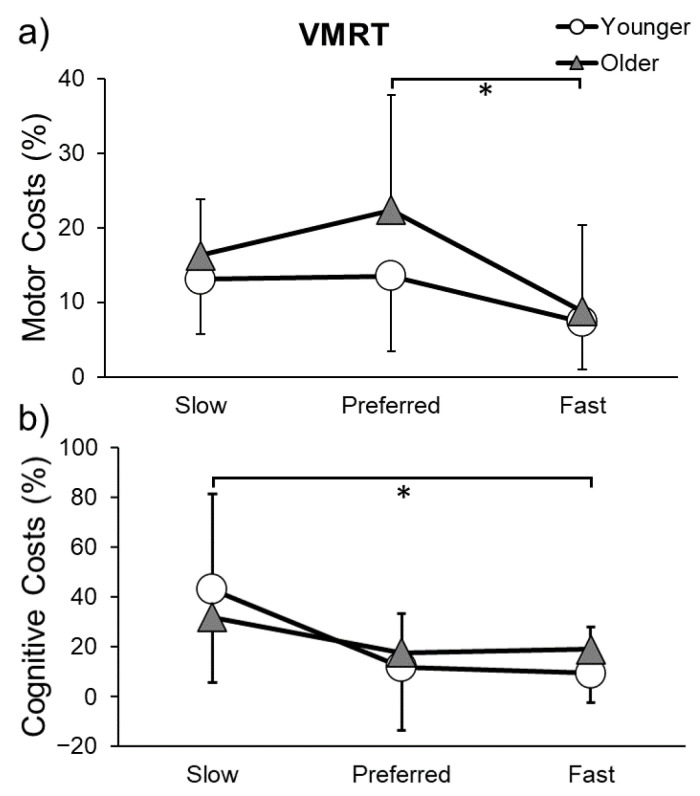
Motor (**a**) and cognitive costs (**b**) while performing the Visuomotor Reaction Time (VMRT) task and walking at slow, preferred, or fast speeds in younger and older adults. * indicates a significant difference (*p* ≤ 0.05) in costs between gait speeds.

There was a significant task × speed interaction for gait speed motor costs (F(6,108) = 18.132, *p* < 0.001, η^2^p = 0.502), step length motor costs (F(6,108) = 11.300, *p <* 0.001, η^2^p = 0.388), and cognitive costs (F(6,108) = 6.978, *p* < 0.001, η^2^p = 0.304). Motor and cognitive costs for the VMRT can be seen in Figure 4. Gait speed motor costs were higher at preferred speed compared to fast speed (*p* = 0.011), and cognitive costs were higher at slow speed compared to fast speed (*p* = 0.030) for both young and older adults. Thus, cognitive and motor performance were less affected by dual tasking when walking fast.

**Figure 5:**
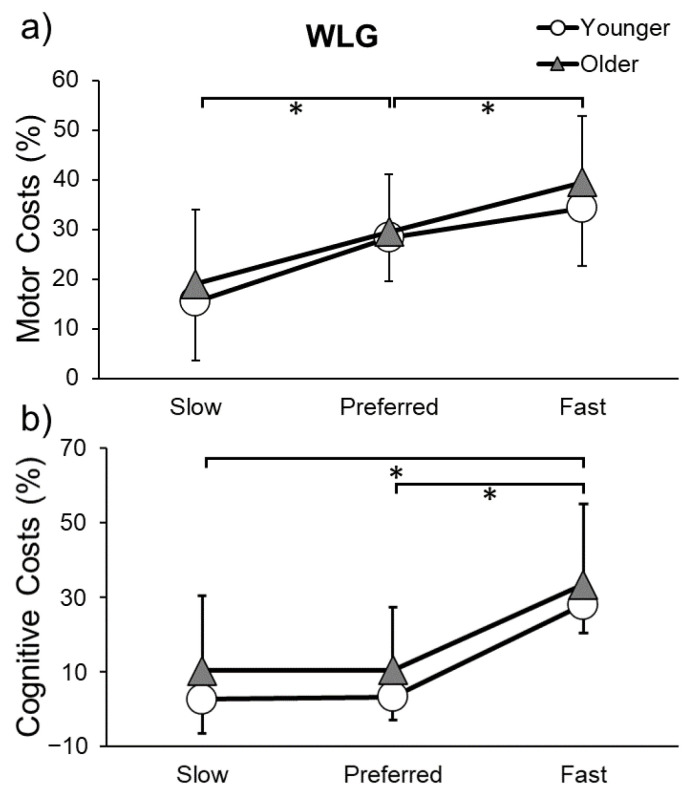
Motor (**a**) and cognitive costs (**b**) while performing the word list generation (WLG) task and walking at slow, preferred, or fast speeds in younger and older adults. * indicates a significant difference (*p* ≤ 0.05) in costs between gait speeds.

Motor and cognitive costs for the WLG can be found in Figure 5. Both gait speed and step length motor costs were higher at fast speed (speed: *p* < 0.001; step length: *p* < 0.001) and preferred speed (speed: *p* < 0.001; step length: *p* = 0.001) compared to slow speed. Cognitive costs were higher at fast speed compared to slow speed (*p* = 0.001) and preferred speed (*p* = 0.008). Thus, there was a larger negative effect of dual tasking on cognitive and motor performance when walking faster.

**Figure 6:**
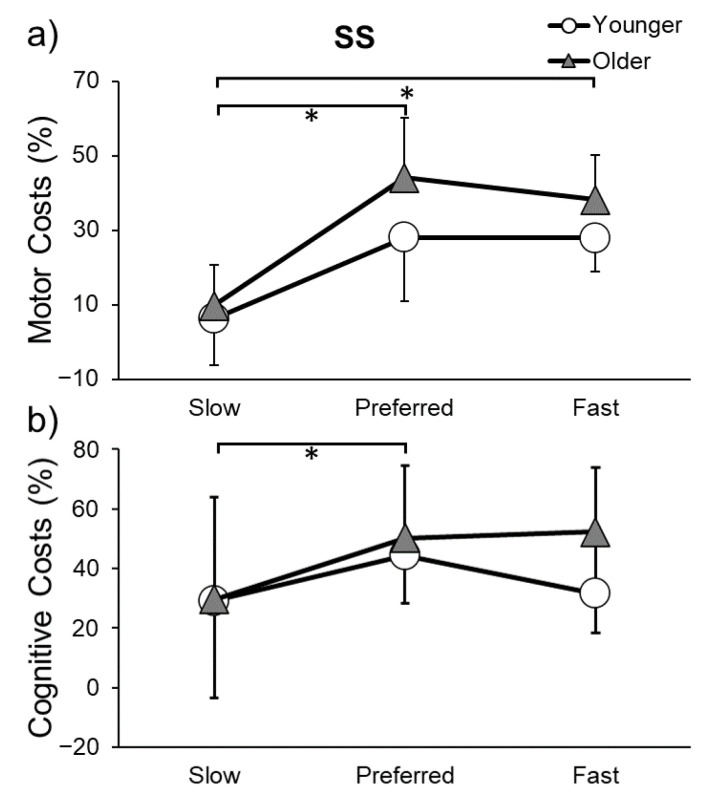
Motor (**a**) and cognitive costs (**b**) while performing the serial subtraction (SS) task and walking at slow, preferred, or fast speeds in younger and older adults. * indicates a significant difference (*p* ≤ 0.05) in costs between gait speeds.

Motor and cognitive costs for the SS can be seen in Figure 6. Gait speed and step length motor costs were higher at preferred speed (speed: *p* = 0.012; step length: *p* = 0.009) and fast speed (speed: *p* < 0.001; step length: *p* < 0.001) compared to slow speed. Cognitive costs were lower in slow speed compared to preferred speed (*p* = 0.045). Thus, dual tasking had a larger negative effect on cognitive and motor performance when walking faster.

**Figure 7:**
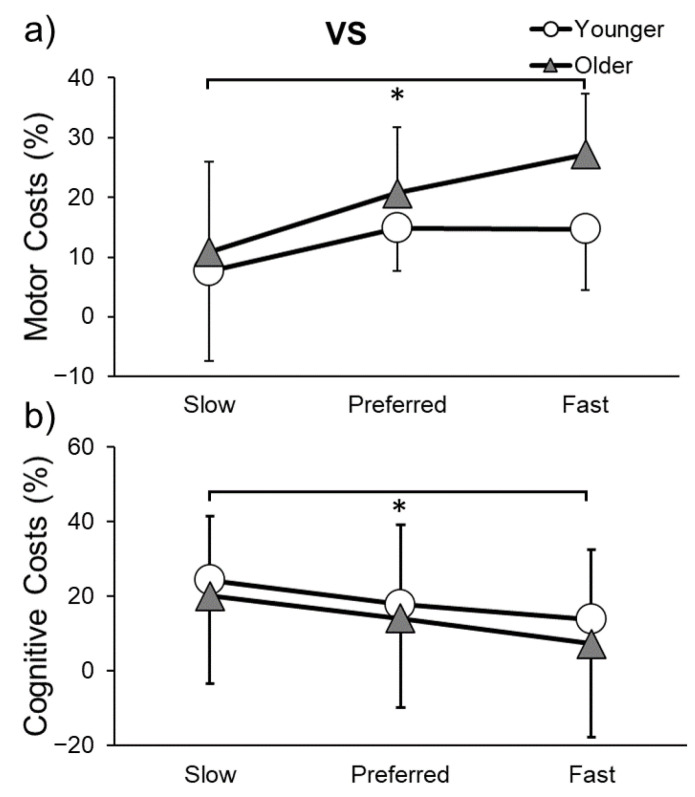
Motor (**a**) and cognitive costs (**b**) while performing the visual Stroop (VS) task and walking at slow, preferred, or fast speeds in younger and older adults. * indicates a significant difference (*p* ≤ 0.05) in costs between gait speeds.

Motor and cognitive costs for the VS can be found in Figure 7. Gait speed motor costs were higher at fast compared to slow speed (*p* = 0.013) and step length motor costs were higher at preferred compared to slow speed (*p* = 0.011). Cognitive costs were higher at slow compared to fast speed (*p* = 0.024). Thus, dual-tasking affected motor performance more at fast speeds, although affected cognitive performance more at slow speeds.

## 4. Discussion

This study evaluated the effects of type of cognitive task, gait speed, and age on CMI during gait. Our first hypothesis was partially supported. Motor costs were larger for WLG and SS compared to VMRT and VS, indicating that there was a larger reduction in gait speed and step length (from single-task walking) while performing these tasks. Cognitive costs were higher for SS than WLG, indicating that there was a larger reduction in cognitive performance from single-task to dual-task for SS. Further, gait speed had a different effect on CMI pattern depending on the type of cognitive task. In line with our second hypothesis, motor and cognitive costs increased as speed increased for most cognitive tasks. However, the opposite relationship between speed and dual-task costs was seen for the VMRT; motor and cognitive costs decreased as speed increased. Lastly, older adults had higher motor costs than young adults, which supported our third hypothesis, although task and speed had the same effects on both age groups.

Both younger and older adults had higher motor costs when performing WLG and SS compared to VMRT and VS, which is in line with our previous findings in young adults [7]. These results indicate that the WLG and SS interfered the most with gait and led to a larger slowing of gait speed and reduction in step length than other tasks. Dual-task theories such as the capacity sharing theory suggest that two tasks will interfere with each other if they share common, overlapping neural resources [36], leaving limited resources for optimal performance on both tasks [37]. Thus, the cognitive domains involved in the WLG and SS (working memory, language/verbal skills, and problem solving) may have highly overlapped with the cognitive domains involved during gait.

In support of this, functional neuroimaging studies have shown that similar brain regions are activated during both working memory tasks and gait, specifically the supplementary motor area and other frontoparietal regions [38,39]. During dual tasking, these neural resources may be devoted toward the cognitive task, leaving few resources available to focus on gait. Further, dual-tasking had a larger negative effect on cognitive performance for the SS than the WLG, suggesting that mental math tasks may interfere the most with both motor and cognitive performance during dual-task gait.

Gait speed also significantly affected dual-task costs for both younger and older adults. Generally, motor and cognitive costs were lower at slow speeds and higher at fast speeds. This indicates that there was a smaller difference between dual-task and single-task performance when walking slowly, which appears to facilitate the ability to focus on both the motor and cognitive tasks. Previous studies have found that slow walking improves control over natural movements of the trunk in the anterior–posterior direction and attenuates changes in joint angles, thereby improving stability [13,40,41,42,43,44]. Additionally, shorter step lengths can improve stability by keeping the center of mass closer within the stability bounds of the base of support [40]. We have not directly measured fall risk and stability and, therefore, cannot correlate those with study outcomes. However, we postulate that slow walking could increase gait stability and reduce CMI by providing more time and available neural resources to process both tasks. By this logic, motor costs could have increased at fast speeds because individuals slowed their gait speed or reduced their step length during dual-tasking to try and increase their resources available to process both tasks and maintain cognitive performance.

The opposite relationship between gait speed and CMI was observed on the VMRT, for which there was a larger difference between single-and dual-task performance at slow speeds. This finding is in line with evidence from previous studies that performance on reaction time tasks is deteriorated by walking slowly. For instance, when verbally responding to an auditory cue, young adults showed increased reaction time at slower walking speeds [45,46,47]. The results can possibly be explained by the conflict in instructions between walking slowly on the gait speed task and responding quickly on the VMRT. Changes in gait speed could have also altered optic flow, contributing to improved reaction time during fast walking or possible priming of the motor system driven by fast gait speed. However, we are limited by the scope of this study to ascertain any of these causal mechanisms.

Cognitive task and gait speed had the same effect on motor costs whether assessing gait speed or step length. This may have occurred because gait speed and step length are biomechanically related, and a reduction in gait speed could be attributed to walking with a smaller step length [48]. However, other spatio-temporal gait parameters can also contribute to gait speed (e.g., cadence). Therefore, we cannot conclude that the changes in gait speed observed in dual-task conditions were necessarily dependent on changes in step length. It is possible that both parameters could have been modulated independently based on the presented task and the amount of available cognitive resources.

Our results indicate that CMI during dual-task gait depends on the unique combination of the cognitive demand of a task and gait speed. Fast walking increases CMI on challenging cognitive tasks which require working memory, mental math, or language skills, but facilitates performance on more simple cognitive tasks which require rapid motor responses. A similar relationship between cognitive demand and CMI has been explained using a U-shaped function during postural control [49,50,51], in which the cognitive demand of the secondary task determines if postural control will be enhanced or diminished. For example, postural sway decreased compared to single-task standing during a relatively easy cognitive task (i.e., viewing digits), whereas postural sway increased during more difficult cognitive tasks (e.g., n-back) [51]. Thus, it may be similarly true that the modulatory effect of gait speed on CMI depends on the cognitive demand of the task.

The increase in CMI observed during specific cognitive tasks (WLG and SS) and at fast gait speeds may have occurred because these conditions required increased brain activity in areas shared by gait. Several neuroimaging studies have shown that gait results in widespread brain activation (for review, see [52]) including areas in the frontoparietal cortex that are associated with attention, executive function, and working memory [53]. Specifically, many studies have reported that the prefrontal cortex is activated while walking [54] and postulate that it is involved in regulating successful locomotion [55]. Further, prefrontal cortex activation occurs when performing WLG and SS tasks [56] and increases when walking faster [57,58]. Performing these cognitive tasks while walking may exceed the capacity of neural resources in the prefrontal cortex and explain why we found a deterioration in performance, especially at increased gait speeds.

Further, age-related changes in activation of the prefrontal cortex could explain why older adults exhibited more CMI than younger adults. Older adults have more activation in the prefrontal cortex than young adults during single-task gait and thus may rely more on neural involvement while walking [59,60]. However, with the addition of a cognitive task, one study reported that older adults conversely had lower prefrontal cortex activity than younger adults [61]. Therefore, it is possible that during dual-tasking, older adults are not able to properly activate the brain areas necessary to maintain gait or cognitive performance, which results in higher CMI.

Our results provide valuable insight into the development of dual-task paradigms for clinical implementation, which has been primarily limited by the lack of protocol recommendation/standardization [16]. Our results indicated that WLG and SS tasks at fast walking speeds induced the highest amount of CMI in older adults. Thus, dual-task assessments may benefit from including similar mental math, verbal fluency, or working memory tasks. Tasks which highly compete with the neural resources required for gait might be more sensitive in identifying individuals that are highly susceptible to CMI and thus may be at a higher risk of motor or cognitive decline.

Including cognitive tasks in addition to gait rehabilitation/training comes at no additional cost or time burden, yet potentially yields larger improvements in gait due to increased training difficulty. Further, incorporating dual-task conditions into gait rehabilitation could also provide the benefit of maintaining or improving cognitive function. On the other hand, cognitive rehabilitation paradigms also could benefit from the addition of motor tasks to simultaneously train cognitive and motor function in challenging conditions. Clinicians should consider incorporating dual-task conditions into existing training protocols to provide a higher impact on patient function and quality of life, with the focus of the training (improving cognitive or motor function) driven by the individual needs and goals of the individual patient.

Further, from a safety perspective, clinicians may want to initiate dual-task assessments or interventions by walking slowly to reduce CMI and gradually progress towards more challenging conditions by increasing gait speed. Additionally, our study showed that it is imperative to consider the interaction between cognitive task and gait speed when administering dual-task assessments. Lastly, our study provided preliminary evidence that a variety of cognitive tasks can interfere with gait, which suggests that many cognitive–motor tasks completed during activities of daily living may induce CMI in older adults. Therefore, clinicians may need to assess multiple combinations of cognitive tasks while walking in order to precisely identify which cognitive domains are deteriorated in older adults.

This study had some limitations which should be taken into consideration for the planning of future research studies. First, although we examined the effect of different gait speeds on dual-task costs, we did not examine how other walking conditions (e.g., perturbed walking, obstacle crossing, narrow walking, and treadmill walking) could affect CMI. Future studies should examine how gait speed, cognitive task, and age could affect dual-task gait in conditions that provide additional challenges to stability. Additionally, we only provided 10 s for participants to complete the WLG, SS, and VS tasks in each trial to prevent adaptation. This brief amount of time may have caused participants to feel rushed and prevented them from optimal performance. Future studies may want to consider using longer durations of dual-task trials. It should also be considered that we needed uniformity of values to compare performance between conditions; thus, we calculated motor and cognitive costs instead of comparing the raw values for gait speed, step length, and cognitive performance.

Additionally, future studies may want to expand their study population and sample size. We included healthy older adults who were high functioning, independently ambulatory, and had intact cognition. Many older adults face losses to physical function (e.g., frailty, balance impairments) and cognitive function (e.g., mild cognitive impairment, Alzheimer’s Disease) that may result in different effects of task and gait speed on CMI. Further, we only included 10 participants in each group, which is a relatively small sample size. However, our a priori power analysis indicated that our study was adequately powered and we found moderate to large effect sizes. Future larger-scale studies would still need to be conducted with an increased sample size to validate our results.

## 5. Conclusions

This study found that the type of cognitive task, gait speed, and age can all affect CMI during dual-task gait. Dual-tasking resulted in the largest reduction in gait speed, step length, and cognitive performance (compared to single-task performance) when performing a working memory task and walking at a faster-than-preferred pace. Therefore, future researchers and clinicians may consider implementing these conditions into dual-task paradigms to improve the accuracy of motor and cognitive status assessment. Future researchers may also consider examining how CMI during dual-task gait could additionally be affected by motor or neurological conditions that lead to balance impairments and increase fall risk.

## Figures and Tables

**Table 1:** Demographic information.

Characteristics	Younger Adults (Mean ± SD)	Older Adults (Mean ± SD)
Age (y)	23.0 ± 2.1	62.5 ± 6.5
Sex (M/F)	5/5	5/5
Weight (kg)	65.5 ± 10.2	90.3 ± 18.0
Height (cm)	165.1 ± 9.1	170.6 ± 11.6
MoCA	-	28.3 ± 1.16
6MWT (m)	-	434.3 ± 137.8

Abbreviations: MoCA = Montreal Cognitive Assessment; 6MWT = 6 min walk test.

**Table 2:** Cognitive scores (Mean *±* SD) on the visuomotor reaction time (VMRT), word list generation (WLG), serial subtraction (SS), and visual Stroop (VS) tasks while standing (single-task) and walking at slow, preferred, and fast speeds (dual-task) in younger and older adults. Cognitive scores are presented as milliseconds on the VMRT and the number of correct responses on the WLG, SS, and VS.

Task	Condition	Cognitive Scores	
		Young Adults	Older Adults
VMRT	Single-Task	330 ± 50	370 ± 100
	Dual-Task		
	Slow	470 ± 150	490 ± 130
	Preferred	390 ± 100	430 ± 130
	Fast	440 ± 330	440 ± 150
WLG	Single-Task	6.29 ±1.76	5.33 ±1.44
	Dual-Task		
	Slow	5.82 ± 2.19	4.56 ± 1.07
	Preferred	5.82 ± 2.24	4.67 ± 1.17
	Fast	4.71 ± 2.82	3.50 ± 1.01
SS	Single-Task	4.47 ± 1.66	5.11 ± 3.74
	Dual-Task		
	Slow	2.94 ± 1.39	4.28 ± 3.25
	Preferred	2.41 ± 1.28	2.72 ± 1.87
	Fast	2.65 ± 1.27	2.33 ± 2.10
VS	Single-Task	14.59 ± 3.06	12.83 ± 1.16
	Dual-Task		
	Slow	11.06 ± 2.88	9.83 ± 3.26
	Preferred	11.94 ± 2.59	10.72 ± 3.66
	Fast	13.19 ± 2.76	11.61 ± 3.64

**Table 3:** Gait speed and step length (Mean ± SD) for young and older adults at their slow, preferred, and fast pace while walking (single-task) and completing four different cognitive tasks (dual-task).

Speed	Condition	Gait Speed (m/s)		Step Length (/bh)	
		Young Adults	Older Adults	Young Adults	Older Adults
Slow	Single-Task	0.76 ± 0.14	0.70 ± 0.11	0.31 ± 0.05	0.30 ± 0.03
	Dual-Task				
	VMRT	0.65 ± 0.13	0.59 ± 0.10	0.28 ± 0.04	0.25 ± 0.04
	WLG	0.63 ± 0.12	0.55 ± 0.10	0.30 ± 0.03	0.27 ± 0.05
	SS	0.68 ± 0.10	0.62 ± 0.12	0.28 ± 0.05	0.28 ± 0.04
	VS	0.68 ± 0.12	0.62 ± 0.15	0.29 ± 0.04	0.28 ± 0.05
Preferred	Single-Task	1.18 ± 0.20	1.03 ± 0.13	0.40 ± 0.05	0.36 ± 0.04
	Dual-Task				
	VMRT	1.02 ± 0.17	0.79 ± 0.19	0.36 ± 0.06	0.30 ± 0.06
	WLG	0.84 ± 0.14	0.71 ± 0.14	0.32 ± 0.06	0.29 ± 0.04
	SS	0.85 ± 0.21	0.57 ± 0.18	0.33 ± 0.05	0.30 ± 0.05
	VS	1.00 ± 0.15	0.82 ± 0.19	0.36 ± 0.05	0.31 ± 0.05
Fast	Single-Task	1.69 ± 0.30	1.43 ± 0.24	0.48 ± 0.07	0.42 ± 0.05
	Dual-Task				
	VMRT	1.55 ± 0.23	1.28 ± 0.23	0.44 ± 0.07	0.38 ± 0.05
	WLG	1.11 ± 0.26	0.85 ± 0.22	0.40 ± 0.08	0.32 ± 0.07
	SS	1.21 ± 0.26	0.88 ± 0.22	0.38 ± 0.08	0.33 ± 0.06
	VS	1.41 ± 0.27	1.02 ± 0.20	0.44 ± 0.07	0.37 ± 0.05

bh: body height; SS: serial subtraction; VMRT: visuomotor reaction time; VS: visual Stroop; WLG: word list generation.

## Data Availability

The data presented in this study are available on request from the corresponding author.
